# Programmed cell death can increase the efficacy of microbial bet hedging

**DOI:** 10.1038/s41598-017-18687-y

**Published:** 2018-01-18

**Authors:** Eric Libby, William W. Driscoll, William C. Ratcliff

**Affiliations:** 1 Santa Fe Institute, Santa Fe, NM 87501 USA; 2 Ecology, Evolution and Behavior, University of Minnesota, Minneapolis, MN 55108 USA; 3 School of Biological Sciences, Georgia Institute of Technology, Atlanta, GA 30332 USA

## Abstract

Programmed cell death (PCD) occurs in both unicellular and multicellular organisms. While PCD plays a key role in the development and maintenance of multicellular organisms, explaining why single-celled organisms would evolve to actively commit suicide has been far more challenging. Here, we explore the potential for PCD to act as an accessory to microbial bet-hedging strategies that utilize stochastic phenotype switching. We consider organisms that face unpredictable and recurring disasters, in which fitness depends on effective phenotypic diversification. We show that when reproductive opportunities are limited by carrying capacity, PCD drives population turnover, providing increased opportunities for phenotypic diversification through stochastic phenotype switching. The main cost of PCD, providing resources for growth to a PCD(−) competitor, is ameliorated by genetic assortment in spatially structured populations. Using agent -based simulations, we explore how basic demographic factors, namely bottlenecks and local dispersal, can generate sufficient spatial structure to favor the evolution of high PCD rates.

## Introduction

Programmed cell death (PCD) describes a genetically encoded process of cellular suicide that is often used as an umbrella term for more specific cell-death phenotypes (e.g., apoptosis, paraptosis, autophagy, chromatolysis, etc.)^1–5^. Anatomists first observed PCD in the context of animal development during the 19th century^4^. Since then, a vast body of literature has established the key role of PCD in both the generation^6,7^ and maintenance of multicellular forms^1,8^. Interestingly, PCD appears to be widespread among distantly related unicellular organisms^9–16^. The origin and maintenance of PCD within multicellular taxa has a straightforward evolutionary explanation if the death of some cells provides a benefit to the organism as a whole. In contrast, the evolution of PCD in unicellular organisms presents a conundrum: under what conditions (and by what mechanisms) will natural selection favor organismal suicide?

Different mechanisms have been proposed to explain the existence of PCD among unicellular taxa^15^. One category of hypotheses proposes that PCD is an altruistic trait favored by kin or multilevel selection, conceptually similar to the evolution of reproductive altruism in organisms such as eusocial insects. These hypotheses propose that PCD may have evolved to remove cells weakened by deleterious mutations, pathogens, or age-accumulated damage^17–22^. Removing such cells improves the health of other members of the population either by preventing the spread of pathogens or making more resources available to healthier cells, akin to the altruistic suicide of parasitized aphids that die rather than let close relatives be infected^23^. Another view is that PCD may have evolved in microbes because of its role in multicellular development. For example, PCD by a subset of a bacterial population may be necessary to provide extracellular DNA that plays a structural role in biofilm formation^24^. Finally, PCD may arise as a pleiotropic side-effect of genes under positive selection because of their pro-survival effects^15^. This would imply that there is no direct adaptive benefit to PCD and its negative effects are simply a tolerable side-effect of a beneficial pleiotropic trait. Although cell death would appear ‘programmed’ in such cases, this hypothesis does not invoke benefits above the cell-level. Unfortunately, few of these potential evolutionary explanations have been experimentally tested or mathematically modeled, and little is known about the ecological conditions necessary for their evolution.

In this paper, we propose a novel evolutionary hypothesis for the maintenance of PCD in unicellular organisms: PCD serves as an accessory to microbial bet hedging. Bet-hedging strategies can be advantageous when populations that lack the capacity to rapidly sense and respond to environmental change within a single generation are subject to fluctuating selection for alternative phenotypes. Such strategies are therefore associated with unpredictable environments and traits that cannot be dynamically regulated (e.g. timing of seed germination in desert annuals). Bet-hedging traits operate in two possible ways. First, they can spread risk among multiple phenotypes, each of which is well-suited to a possible future environment (diversification bet-hedging)^25,26^. Second, they can allow organisms to pursue a generalist strategy that performs acceptably across a range of possible future environments (conservative trait bet-hedging)^27^. Of the two types of bet-hedging, most of the well-established traits act as diversification bet-hedging, but this may be because it is more conspicuous than conservative trait bet-hedging^28^. Micro-organisms typically enact diversification bet-hedging strategies through stochastic phenotype switching, in which reproducing cells give rise to phenotypically distinct offspring with a low (typically 10^−1^ to 10^−5^) probability^29,30^. Since the offspring can switch back to the original phenotype at some low probability, stochastic phenotype switching typically generates bistable populations in which a single genotype exhibits two distinct phenotypes^31,32^. In contrast to mechanisms of phenotypic regulation that operate within generations (e.g. transcriptional regulation), these examples of stochastic phenotype switching require generational turnover to create variation. Even at relatively high rates of switching (10^−3^), it still takes more than 1,000 generations for an initially uniform population to reach maximum levels of phenotypic diversity^29^.

Here we examine the conditions under which PCD increases the efficacy of microbial bet-hedging by creating generational turnover, resulting in increased phenotypic diversity. We analytically examine the co-evolution of PCD and stochastic phenotype switching in an unpredictable environment in which more diversified populations have higher long-term fitness. Because population size in our model is constrained by a carrying capacity, PCD allows organisms to circumvent this limitation to reproduction. As organisms die, they create opportunities for others to reproduce and diversify via stochastic phenotype switching. Thus PCD incurs both costs and benefits: some cells die, but if surviving clonemates can use spared resources to divide, then the genotype as a whole will become more diversified.

One possible downside of this strategy is that the resources made available by PCD are susceptible to exploitation by PCD− competitors. We show that across many cycles of unpredictable environmental risk, exploitation by PCD− competitors does not necessarily overwhelm the long-term fitness advantage gained by the more diversified PCD+ strain. Further, the cost of PCD is highly dependent on the degree of population structure. When individuals that die are more often replaced by nearby clonemates than unrelated competitors, the cost is reduced. More importantly, we find that the conditions required for selection to favor elevated PCD in our model are very permissive: elevated PCD can evolve in microbes with a wide range of stochastic switching frequencies, in environments with a wide range of disaster frequencies, and in populations with modest spatial structuring. We contextualize these results using a spatially explicit biofilm simulation. We find that, even if the simulation is initiated with a well-mixed population, the dynamics of occasional environmental catastrophe followed by range expansion generates sufficient levels of spatial structure to favor PCD. These results point to new adaptive explanations for the evolution and maintenance of PCD in unicellular populations by focusing attention on the profoundly non-equilibrium nature of many microbial populations (particularly those exploiting patchily-distributed, ephemeral resources).

## Methods

We consider a competition between two microbial strains (*G*_*p*_ and *G*_*n*_) in an environment that experiences frequent disasters. The *G*_*p*_ strain has programmed cell death (PCD+) while the *G*_*n*_ strain does not (PCD−). Each strain exhibits two possible phenotypes: *A* and *B*. We denote the number of organisms of phenotypes *A* and *B* of the *G*_*p*_ genotype as *A*_*p*_ and *B*_*p*_ and similarly *A*_*n*_ and *B*_*n*_ correspond to the phenotypes of *G*_*n*_. The only meaningful difference between *A* and *B* phenotypes is their susceptibility to an environmental disaster. Disasters occur randomly and target a single phenotype for annihilation–in this way they are similar to other disasters that microbes might face such as antibiotic exposure, infection by lytic phage, or immune system recognition. Following a disaster, the surviving types reproduce until the total population is restored to a carrying capacity, *N*. Phenotypic diversification occurs via stochastic phenotype switching such that each time a strain reproduces there is a probability *p* that the alternate phenotype will be produced. In between disaster events, there are rounds of PCD in which the *G*_*p*_ strain loses cells according to its characteristic rate of PCD determined by *c*. The population is restored to *N* in a manner that depends on the spatial arrangement of the population, characterized by the structure parameter *r* ∈ [0,1]. *r* determines the probability that a cell dying from PCD will be replaced by growth from the same genotype. When microbes are growing in highly structured populations, such that the two genotypes rarely intermix, *r* will be near 1 and most cells dying from PCD will be replaced by living clonemates, not competitors. *r* is a dynamic feature of the population, changing in response to death (both PCD and that caused by environmental disaster), growth, and cellular migration. To this end, we analyze three versions of our basic underlying model, each with increasing complexity. We build up from a simple mathematical model holding *r* fixed (which is the least realistic but offers the clearest insight into the importance of *r* in the evolution of PCD) into a 3D biofilm simulation within which *r* emerges naturally from the cell-level demographic processes of birth, death, and migration.

**Figure 1:**
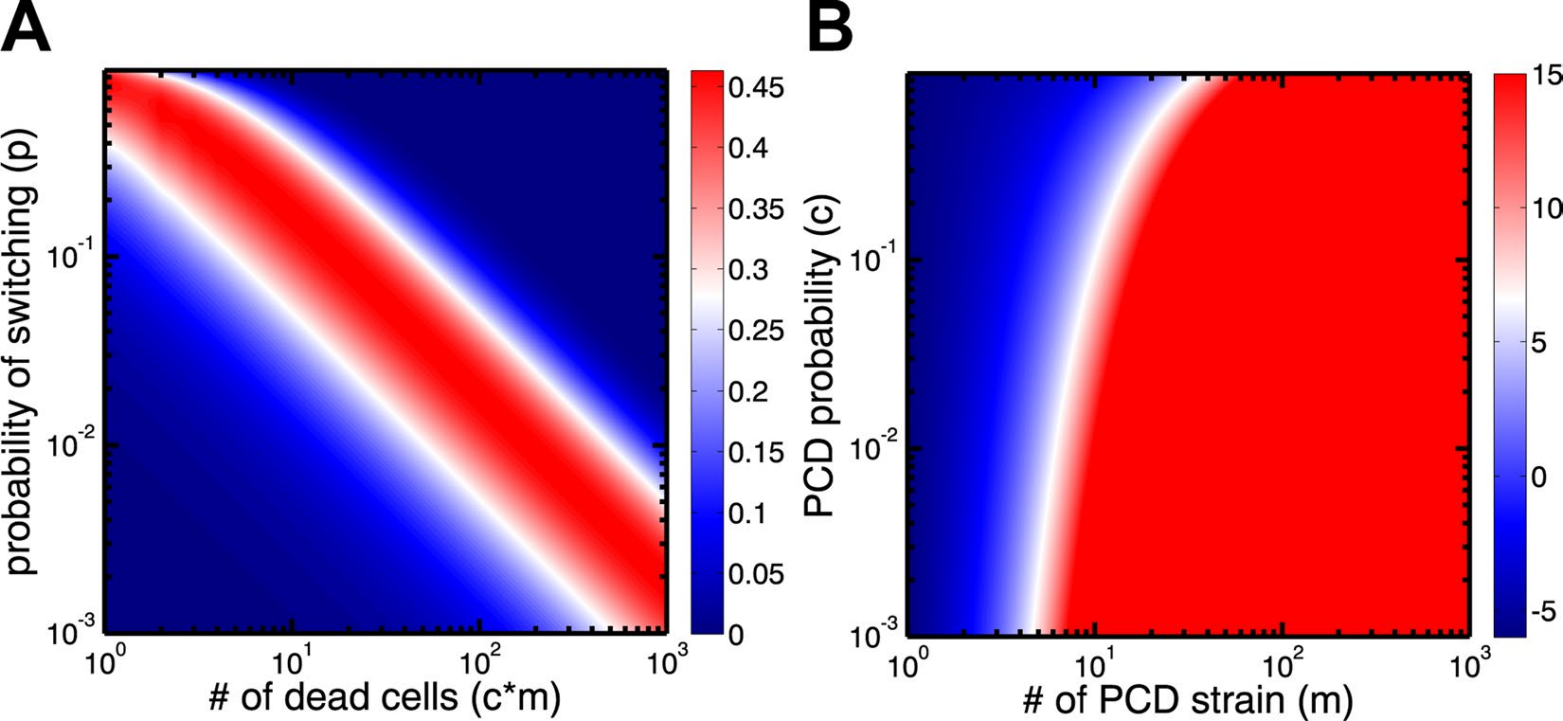
Cost and benefit of PCD. (**A**) A contour plot of the benefit of PCD from Eq. 3 for a range of switching probabilities (*p*) and numbers of PCD cells (*c* * *m*) when *r* = 0.75. There is a narrow band where PCD is beneficial. Outside of this area, the switching rate is too high or too low to incur a benefit to PCD+ genotypes. (**B**) A contour plot shows the *log*_10_ relative benefit versus cost of PCD as expressed in Eq. 5 for a range of PCD probabilities (*c*) and number of cells (*m*) with *r* = 0.5 and *p* = 10^−6^. The blue area corresponds to a greater cost while the red area corresponds to a greater benefit. Since the plot is transformed by *log*_10_ the benefit is many orders of magnitude (>10) greater when the number of cells is larger than 10.

### Spatially-implicit model with fixed *r*

In our first model, we consider a simple deterministic description of the dynamics in between disasters, assuming a fixed *r* and a population at carrying capacity. The equations in Eqn. 1 describe these dynamics for a single round of PCD and repopulation. We analyze these equations to determine the expected costs and benefits of PCD and predict the conditions that might favor it. We then use stochastic simulations to consider the cumulative effect of many discrete rounds of PCD/repopulation and disasters. In these simulations we treat the switching (*p*), PCD (*c*), and structure (*r*) parameters as probabilities (still holding *r* fixed). We simulate the population dynamics over the course of ten thousand discrete rounds of PCD and disasters or until a genotype goes extinct. A genotype is said to “win” if the opposing strain goes extinct first or if at the end of ten thousand rounds it makes up a higher percentage of the total population.

1 $$\begin{array}{rcl}{A}_{p}(t+\mathrm{1)} & = & {A}_{p}(t)-c{A}_{p}(t)+cr\mathrm{(1}-p){A}_{p}(t)+prc{B}_{p}(t)\\ {B}_{p}(t+\mathrm{1)} & = & {B}_{p}(t)-c{B}_{p}(t)+cr\mathrm{(1}-p){B}_{p}(t)+prc{A}_{p}(t)\\ {A}_{n}(t+\mathrm{1)} & = & {A}_{n}(t)+c({A}_{p}(t)+{B}_{p}(t\mathrm{))(1}-r)\\  &  & \times (\frac{{A}_{n}(t)}{{A}_{n}(t)+{B}_{n}(t)}\mathrm{(1}-p)+\frac{{B}_{n}(t)}{{A}_{n}(t)+{B}_{n}(t)}p)\\ {B}_{n}(t+\mathrm{1)} & = & {B}_{n}(t)+c({A}_{p}(t)+{B}_{p}(t\mathrm{))(1}-r)\\  &  & \times (\frac{{B}_{n}(t)}{{A}_{n}(t)+{B}_{n}(t)}\mathrm{(1}-p)+\frac{{A}_{n}(t)}{{A}_{n}(t)+{B}_{n}(t)}p)\end{array}$$

### Spatially-explicit lattice model with evolving *r*

In our second model, we consider an explicit population structure of a 100 × 100 lattice. We fill the lattice with an equal number of PCD+ and PCD− cells that are assigned an *A* or *B* phenotype according to a probability of 0.5. Cells are organized into initial configurations with different average values of *r* through an iterative process. We use the stochastic dynamics approach from our first model to simulate the population dynamics on the lattice. The key distinction with the first model concerns how cells are replaced following death via PCD or a disaster. In the lattice model, we repopulate the entire community through an iterative process in which dead cells are randomly replaced by a living cell in an adjacent lattice point, i.e. one of 8 possible nearest neighbors for interior lattice points. We iterate this until the lattice is full before exposing the community to another potential disaster or round of PCD. We continue the simulations until a genotype goes extinct or 100,000 rounds have passed.

### Emergence of spatial structure in a 3D agent-based model

Finally, we expand the 2D lattice model to a 3D agent-based model with dispersal and carrying capacities in different subpopulations. The agent-based model consists of cells distributed across patches that represent micro-environments within 3D space. The patches are arranged in a grid and each can hold 10 cells at which point it is at carrying capacity. Cells are mobile agents capable of reproduction and death. As in our other models, there are four types of cells corresponding to two genotypes, each of which produces two phenotypes that are defined by their resistance/susceptibility to disasters. The main difference between genotypes is that cells in one have a chance of undergoing PCD (the PCD+ strain), while the other does not (the PCD− strain).

The  agent-based model progresses via discrete time steps, during which cells and patches update their state variables in random order. Every time step there is a fixed probability that a disaster will occur and eliminate 99% of the susceptible phenotype across all patches. The choice of which phenotype is affected (either *A* or *B*) is determined randomly and without bias. Following the disaster, if there is space available in the patch, cells reproduce and give rise to an alternative phenotype with a constant probability. After reproduction, cells can migrate to adjacent patches if there is room according to a fixed probability which is the same for all cells. At the end of a time step, cells of the PCD+ genotype undergo cell death according to a constant probability. Simulations end when a genotype goes extinct.

### Code and supplementary material

We include computer code for the simulations of each of the three models in the supplementary material. We also include an appendix in which we first explore a continuous time model in which the population is not assured of reaching carrying capacity between disasters (Figures S1 and S2), and an evolutionary simulation in which PCD rates are allowed to evolve via mutation (Figures S3 and S4). In both cases we show that environments with frequent, unpredictable disasters can favor the evolution of PCD.

## Results

### Cost of PCD

We begin our analysis by considering the costs and benefits of PCD within the context of our deterministic model with fixed *r* (see Eqn. 1). There is a direct cost to PCD because cells are killed. If all cells were replaced by the same strain then this would remove the cost. However, if replacement does not occur in completely phase separated populations (i.e. *r* < 1) then some of the cells that die will be replaced by members of the competing strain. We call the total number of a strain replaced by a competing strain the cost of PCD. In our first model, the population remains at carrying capacity until a disaster strikes. Despite being at carrying capacity, PCD and regrowth allow for population turnover. We calculate the expected cost of PCD during the period between disasters using the equations in Eqn. 1. If one strain uses PCD and the other does not then after *t* rounds of PCD/regrowth the PCD+ strain will decrease by a factor shown in Eqn. 2.

2 $${(c(r-\mathrm{1)}+\mathrm{1)}}^{t}$$

This factor has three terms which determine its magnitude: *c*, *t*, and *r*. As expected, the cost of PCD is high for large values of *c*, i.e. the PCD rate. Similarly, if disasters are infrequent, i.e. *t* is large, then the cost is high because it effectively increases the total amount of PCD that occurs in between disasters. Since the *r* parameter determines how many cells that undergo PCD are replaced by the same genotype, higher values of *r* reduce the cost of PCD. When *r* = 1, Eqn. 2 is equal to 1 which means that there is no cost to PCD. A parameter that appears to be missing from the cost factor is the switching rate, *p*. This is because the cost is absorbed by the strain as a whole without regard to how it is partitioned into *A* and *B* types.

### Benefit of PCD

While the cost of PCD is shared by all members of a genotype without regard to phenotype, the benefit of PCD is determined by diversification among phenotypes. In our models, PCD indirectly promotes diversification via stochastic phenotype switching by increasing the number of reproductive events between disasters. Such diversification could manifest in a more equal mix of the population among phenotypes, which can increase long-term fitness by reducing variance in fitness across disasters. Alternatively, if there are few opportunities for diversification between disasters (for example, strains with a very low rate of stochastic switching), higher diversification rates can reduce a strain’s possibility of extinction. We will focus on this second manifestation because it is more crucial to the survival of the PCD+ strain–failure to diversify can result in extinction.

We assume that one phenotype, say *B*, has just succumbed to an environmental disaster and is no longer present in the population. Each strain only exists as the *A* phenotype with *m* members of the PCD+ strain (*A*_*p*_) and *N* − *m* of the PCD− strain (*A*_*n*_). Prior to the next disaster, each strain must produce *B* phenotypes or face extinction should the next disaster target *A* phenotypes. Although there may be several rounds of PCD/regrowth in between disasters, we investigate the consequences of just one round of PCD/regrowth. We calculate the expected number of *B* types produced by each genotype assuming that the switching probability is the same, i.e. *p*. The number of *B* types produced by the PCD+ genotype (*B*_*p*_) is *prcm* and the PCD− genotype (*B*_*n*_) is *p*(1 − *r*)*cm*. The difference between *B*_*p*_ and *B*_*n*_ is determined solely by the *r* parameter. We note that the *B*_*n*_ types produced by the PCD− genotype only happen as a result of PCD undergone by *G*_*p*_ when replacement was not perfectly assortative (*r* < 1).

One advantageous situation for the PCD+ strain would be if it produced a B phenotype but the PCD− strain did not. The probability of this event is shown in Eqn. 3.

3 $${\mathrm{(1}-p)}^{cm\mathrm{(1}-r)}-{\mathrm{(1}-p)}^{cm}$$

The probability increases with the structure parameter *r*, reaching a maximum at *r* = 1. It is highest at *p* = 1 − (1 − *r*)^(1)/(*cmr*)^ which for *r* ≪ 1 corresponds to approximately one expected *B* phenotype produced by the PCD+ strain (see Fig. 1A). At slower switching rates and lower rates of PCD, the PCD+ strain is not likely to diversify. At higher switching rates and higher rates of PCD the PCD− strain is likely to diversify along with the PCD+ strain, thereby reducing the relative advantage of PCD.

The benefit of PCD is expressed in terms of avoiding extinction. For comparison, we can adopt a similar currency for the cost by considering the probability that the PCD+ strain goes extinct because of PCD (shown in Eqn. 4). This requires all *m* cells to undergo PCD and be replaced by the competing strain.

4 $${c}^{m}{\mathrm{(1}-r)}^{m}$$

In considering extinction, the PCD+ strain may diversify when its competition does not but it also faces the probability of going extinct from PCD. The ratio of the probabilities of these two events, in terms of benefit to cost, is shown in Eqn. 5.

5 $$\frac{{\mathrm{(1}-p)}^{cm}\mathrm{((1}-p{)}^{-cmr}-\mathrm{1)}}{{(c\mathrm{(1}-r))}^{m}}$$

The ratio is small when *p* is very high (*p* = 1) or when *p* is very low (*p* ≈ 10^−6^) and *r* is small (*r* ≤ 0.5). If we assume a low *p*, say *p* = 10^−6^, and an unbiased *r* (i.e., that of a well-mixed population, *r* = 0.5), then we can compare the ratio of probabilities for a range of values of *c* and *m* (see Fig. 1B). The potential for PCD to enhance microbial bet -hedging is clear: if there are more than 10 *G*_*p*_ cells in the population and the rate of PCD is low (<0.1), then PCD is more than 6 orders of magnitude more likely to be beneficial rather than costly (Fig. 1B).

### Interplay between cost and benefit

**Figure 2:**
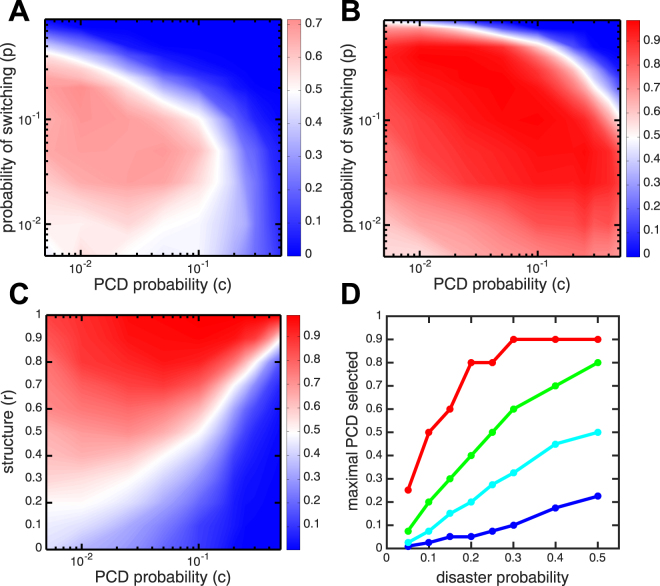
Competitions between PCD+ versus PCD− genotypes. (**A**) Contour plot shows the percentage of wins for the PCD+ strain in competitions where at least one strain survived, over a range of switching probabilities (*p*) and PCD probabilities (*c*) with *r* = 0.5 (an unstructured population) and disaster probability of 0.1. There is an intermediate range of switching probabilities and PCD probabilities where the PCD+ strain is more successful. The blue area corresponds to where PCD+ loses to PCD− cells and the red area is where PCD+ wins. (**B**) Same as (**A**) but with *r* = 0.9 (a highly-structured population). PCD is more successful (higher number of wins) over a larger area of parameter space. (**C**) The success of PCD+ strains versus a PCD− competitor are shown as a function of PCD rate *c* and the assortment parameter *r*. The disaster probability is 0.1 and the switch rate for both genotypes is 0.1. With increasing *r* PCD is more beneficial. (**D**) The maximal amount of PCD selected is shown as a function of disaster probability for different values of *r*: 0.3 (blue), 0.5 (cyan), 0.7 (green), and 0.9 (red). Higher values of PCD are permitted for higher frequencies of disaster where there is greater benefit for diversification and in more structured environments where there is a lower cost to PCD.

While the benefit of PCD can outweigh the cost under some circumstances (Fig. 1B), it is not clear how often those circumstances arise. In a competition, it could be that it is rare for both organisms to only exist in one phenotype after a cycle of a disaster and restoration to carrying capacity. To evaluate how a genotype with PCD fares in competition, we compete a PCD+ strain versus a PCD− strain over a range of switching probabilities (*p*) and PCD rates (*c*), using stochastic simulations (see Supplementary material for computer code). The results shown in Fig. 2A reveal that the PCD+ strain outcompetes the PCD− strain for intermediate switching probabilities when PCD is not too frequent, i.e. *c* < 0.1. For PCD rates above 0.1, the cost is too high to compensate for any benefit. For high switching probabilities, the benefit of PCD is diminished because it is likely that the PCD− strain will always diversify. Similarly, if the switching probability is too low then the PCD+ strain cannot adequately diversify. For more structured environments, with say *r* = 0.9 shown in Fig. 2B, the PCD+ strain wins over a much larger area of parameter space. This is because the higher value of *r* reduces the cost of PCD. If we fix the rate of switching to *p* = 0.1 then we see that as the structure parameter *r* increases, larger values of *c* can be tolerated, i.e. higher PCD genotypes are successful (Fig. 2C,D). In addition, with increasingly frequent disasters, genotypes with higher values of PCD can outcompete PCD− genotypes—the competitive benefit of diversification via PCD outweighs the cost.

**Figure 3:**
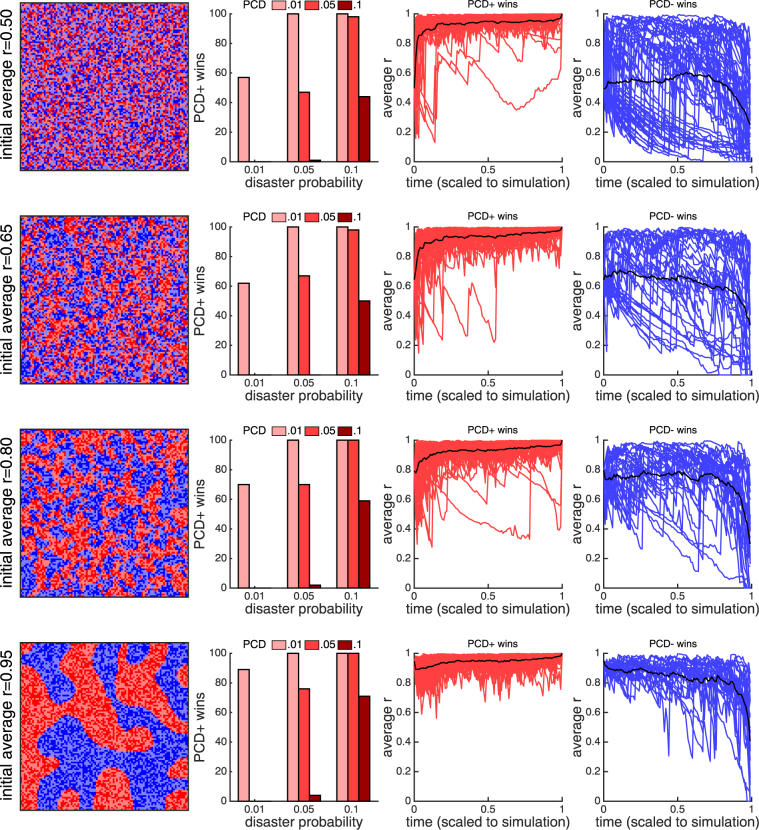
A spatial lattice model with varying *r*. Each row of figures corresponds to a different initial spatial structure organized by the average value of *r*. In each case the leftmost figure shows an example of the initial population state (independently generated for each simulation), where PCD+ genotypes are shown in red (light for *A*_*p*_ and dark for *B*_*p*_) and PCD− are in blue (light for *A*_*n*_ and dark for *B*_*n*_). The bar graphs show the number of times PCD+ genotypes win out of 100 competitions for different probabilities of PCD and disasters. In all cases, higher disaster probabilities allow PCD+ strains to win more often and can support strains with higher rates of PCD (comparing PCD = 0.1 at disaster probabilities of 0.01 and 0.1 is significant with *p* ≪ 0.01 using a Fisher’s exact test). The next two panels show the average value of *r* in simulations that PCD+ won (red) and PCD− won (blue) over time. Time is scaled by the length of the simulation. The disaster probability and PCD probability are both 0.05. The black line shows the mean across the simulations. When PCD+ wins the mean *r* value is high and stays close to 0.9. When PCD− wins, the *r* value is lower and fluctuates more (shown by the variation in the blue plots).

### Spatially-explicit *r*

Until now we have considered a model in which *r* has a fixed value during a simulation. While this assumption allows us to examine how each parameter in the model affects selection for PCD (Fig. 2), it isn’t ecologically realistic. Instead, *r* should change over time as a result of cellular death, reproduction, and changes in overall genotype frequencies. Here we allow *r* to change dynamically, simulating PCD+ and - strains in competition on a 2D surface. Cells occupy different points on a 2D lattice, and when one dies, it is replaced randomly from an adjacent neighbor. While there is no global *r* value (each PCD+ cell has its own *r* which is determined by its local environment), mean values of *r* can be calculated for the PCD+ genotype and should correspond to *r* in the previous model. We consider different initial states of the lattice which describe populations with varying degrees of structure (different average *r* values; see Fig. 3).

Although populations begin with different initial average values of *r*, the results are similar. The time evolution of *r* in simulations tends towards two characteristic forms: either *r* approaches ≈ 0.9 where it remains steady or it experiences large fluctuations and ultimately decreases. Typically, the first scenario results in a win for the PCD+ strain while the second scenario results in a win for the PCD− strain. The key factor in determining which scenario is observed more often is the frequency of disasters. With increased disaster probability the PCD+ strains win more often for a fixed probability of PCD.

In our previous model with fixed *r*, fluctuating disasters (i.e., switching between targeting A and B cells) selected for increased phenotypic diversification, favoring PCD+ strains over PCD− strains. That is still the case in these spatial simulations, but fluctuating disasters provide an additional benefit for PCD+ strains in that they promote high values of *r*. Following a disaster, cells repopulate neighboring empty spaces. This process works to increase spatial structure which in turn lowers the cost of PCD, allowing PCD+ strains to be more competitive with PCD− strains. Across all of our simulations in Fig. 3, we find that in rounds without disasters the average *r* changes by −0.0023, while in rounds with a disaster that switches targets from the previous disaster the average *r* changes by 0.0792. This model reveals a synergy in how environments characterized by frequent disasters select for PCD: they put a premium on phenotypic diversity (increasing the benefit of PCD) and they act to structure the environment (decreasing the cost of PCD).

### Emergence of spatial structure in a 3D model

Our analyses thus far have relied on a notion of spatial structure *r* that determines how much of the resources made available by PCD actually return to the genotype liberating them through programmed cell death. In the first model we held *r* fixed while in the second model we seeded populations with an initial spatial structure and allowed *r* to evolve. In practice, spatial structure is an emergent property of a population which arises from interactions among cells (e.g., birth/death, motility) and population-level processes (e.g., competition, disaster). Here, we use an agent-based model (ABM) to examine the potential for such processes to generate sufficient *r* to favor PCD.

**Figure 4:**
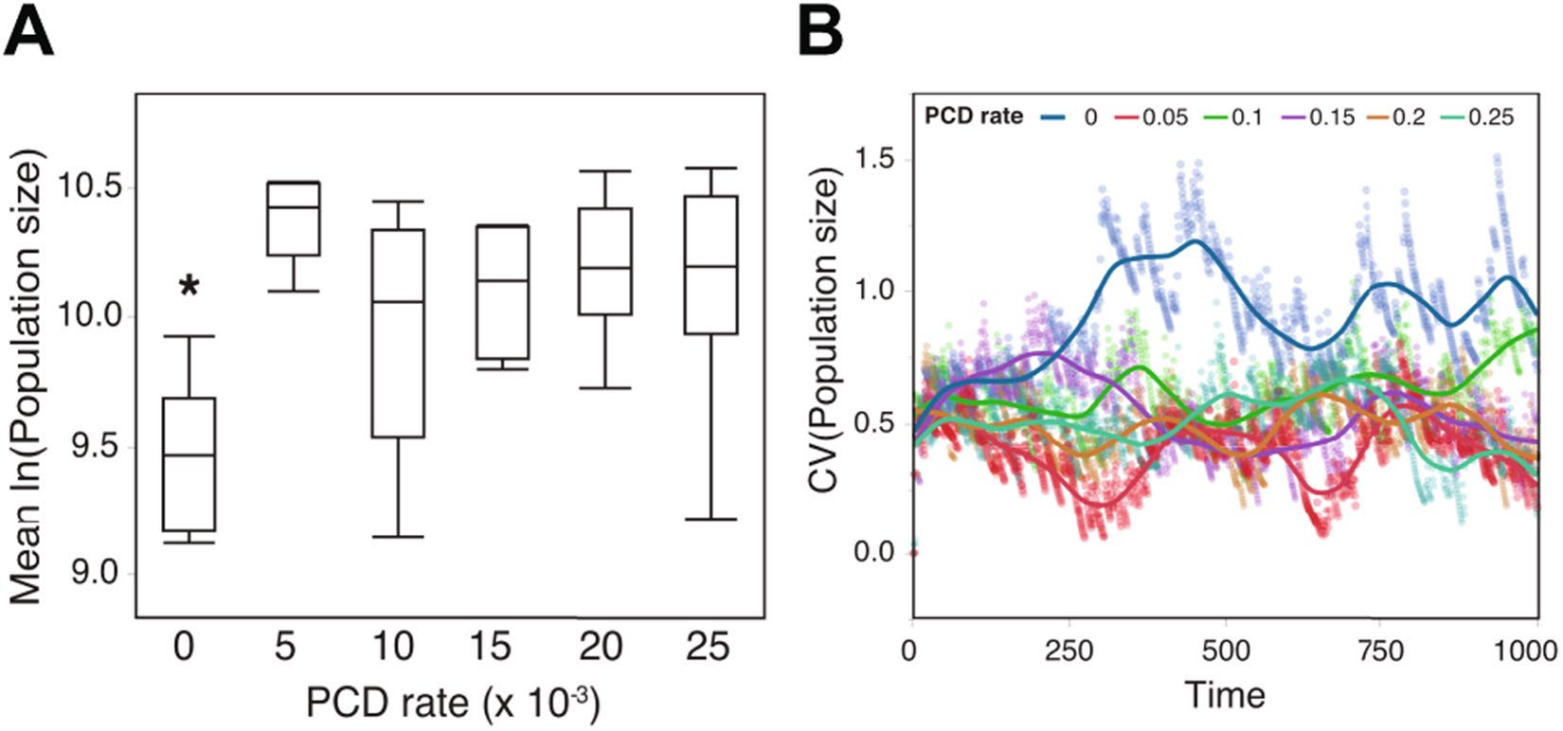
PCD effects in clonal populations in the ABM. (**A**) The grand mean log population size of clonal populations is shown for strains with different amounts of PCD. Populations without PCD are significantly smaller than PCD+ populations (Tukey’s HSD, p < 0.05). Data are from 10 simulations run with a disaster probability of 0.01, migration rates of 0.005 and 0.01 (5 reps each), and for patch-level K of 10. (**B**) The coefficient of variation in population size is shown across time for strains with different amounts of PCD. The PCD+ strains have lower variation than the PCD− strain, indicating that cell death buffers populations from variation in fitness through time.

As with our prior models, PCD was beneficial in the ABM, increasing mean population size (Figure 4a) by accelerating phenotypic diversification, reducing the impact of disasters. In addition, PCD− populations show higher variation in size compared with PCD+ populations over time (Fig. 4b). Although these simulations demonstrate the advantages of PCD in clonal populations, population structure ultimately determines whether the demographic benefits of PCD in monocultures translates into Darwinian advantages in direct competition with a PCD− strain.

**Figure 5:**
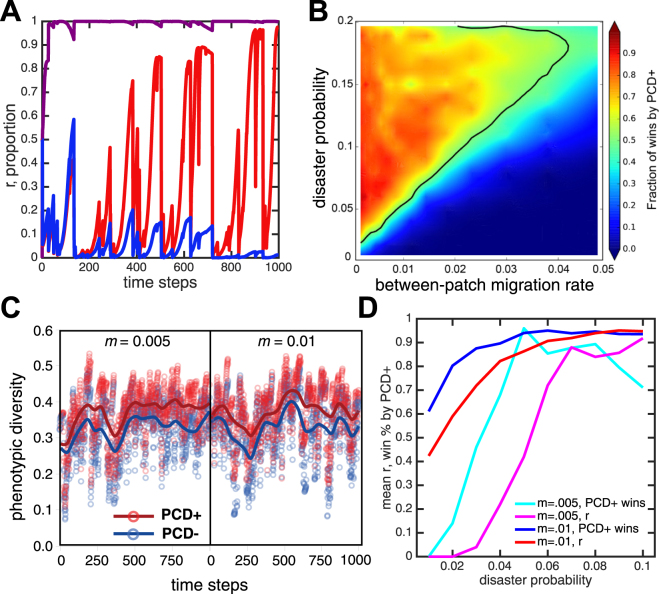
Generation of structure (*r*) via population processes. (**A**) A characteristic time series of an ABM simulation showing the prevalence of PCD+ (red) and PCD− (blue) as a fraction of the global carrying capacity as well as the value of *r* (purple). Populations decrease following disasters and *r* increases during the ensuing recovery. Migration and PCD reduce *r* and the prevalence of the PCD+ strain. (**B**) A contour plot shows the fraction of simulations won by PCD+ as a function of disaster probability and migration. More frequent disasters favor the PCD+ strain, but this is undermined by high rates of between-patch migration. The solid line represents equal competitiveness between PCD+ and − strains. One hundred simulations were run for each of the 340 parameter combinations. (**C**) The phenotypic diversity of PCD+ is consistently higher than PCD− strains across time. PCD+ strains are significantly more diverse than competing PCD− strains (p =< 0.01). Data are from simulations with PCD rates of 0.025 and a disaster frequency of 0.01. (**D**) The mean value of *r* and the fraction of wins by PCD+ are shown for two different migration probabilities. In both cases, *r* and the fraction of wins increases with disasters.

In competitions between PCD+ and PCD− strains, recurring disasters combined with clonal expansion produce sufficient population structure to allow PCD+ strains to outcompete PCD− strains (see Fig. 5a,b). Overall, the relative performance of the PCD+ strain increases with the frequency of disasters. There is an exception at very high disaster probabilities in which the carrying capacity is rarely met and PCD offers few opportunities for increased reproduction. Apart from this exception, frequent disasters select for PCD+ strains. This is primarily because disasters select for rapid phenotypic diversification and PCD increases phenotypic diversity (see Fig. 5c). Moreover, disasters increase the mean value of spatial structure (Fig. 5d), which reduces the net cost of PCD to PCD+ strains. Disasters promote population structure by reducing population size, which has two effects. First, it allows for population expansion into unoccupied patches which increases assortment/structure. Second, it reduces the negative effect of migration on population structure–migration reduces population structure but is most effective at high population densities. As populations recover between disasters, the PCD− strain increases in abundance and the value of *r* decreases (Fig. 5a). This happens because population structure dissipates through cell migration into occupied patches, and also because PCD− cells occasionally replace dead PCD+ cells. This process would ultimately drive PCD+ strains extinct if it were not for disasters, which favor PCD directly by incentivizing phenotypic diversification, and indirectly by generating spatial structure that shields PCD+ strains from competition with PCD− strains.

## Discussion

Recent years have seen a growing awareness that programmed cell death (PCD) may be widespread among unicellular organisms^15,16^, yet few evolutionary hypotheses for its origin have been theoretically or experimentally investigated. Here we propose a novel hypothesis for the evolution of PCD in unicellular organisms: it increases the efficacy of microbial bet-hedging by creating generational turnover that yields increased phenotypic diversity. Using a simple bet-hedging model, we show analytically and computationally that PCD can be adaptive. In our model, PCD is advantageous because it allows microbes to become more phenotypically diversified, which is adaptive in the face of environmental uncertainty. It can be costly, however, if cells that die provide reproductive opportunities for competitors, not kin. In general, we find that fairly high rates of PCD can be favored by selection assuming low rates of stochastic phenotype switching (making population turnover from PCD more valuable) and high levels of genotypic assortment caused by spatial structure in the population (allowing the genotype undergoing PCD to capture most of the resources liberated by cellular suicide). Using an agent-based model we show that high rates of genotypic assortment for PCD+ strains (≈0.9) can emerge through normal demographic processes of reproduction and the bottlenecking effect of environmental disasters.

Due to the seemingly selfless nature of PCD, potential explanations have typically been sought at levels below (e.g. gene-^33^ or phage-level^34^;) and above (e.g. colony-, lineage- or population-level^18,22,35^;) that of the cell. It is also possible that the appearance of evolved PCD may occasionally arise as a byproduct or “malfunction” of traits that may confer cell-level advantages under certain environmental conditions^15^. Extant hypotheses for the adaptive significance of microbial PCD propose that it can provide novel functionality, coordinating “prokaryotic developmental programs”^35,36^, directing resources to mutant lineages that may be more capable of surviving a changing environment^37^, or limiting the spread of pathogens to relatives^38^. Our hypothesis, that PCD can make bet-hedging strategies more effective, is both distinct from these alternatives, and may be fairly general, as stochastic phenotypic diversification is used to hedge against many different forms of risk^29,39–43^.

We note that in order for PCD to be beneficial there are two key requirements. First, reproduction must increase the chance of a phenotypic switch. This can occur through any mechanism tied to progressing through the cell cycle and reproduction. If, instead, cells switch stochastically at any point in time then there is no advantage to diversification via PCD. The second key requirement is that there are limits to cell reproduction, e.g. a carrying capacity. If cells can always reproduce then there is no advantage to progress through a death and replacement step mediated via PCD–diversification can simply occur through more reproduction. However, once reproduction is limited then cells must find new ways to increase diversity.

Why would organisms ever use PCD as a mechanism to diversify, when they could simply evolve faster switch rates? This would allow genotypes to diversify during growth and avoid the costs of cell death. PCD is thus a somewhat inelegant solution, entailing costs that the direct evolution of switch rates would not. We see two scenarios where PCD may be a useful tool. First, if evolving higher switch rates is either difficult or costly, PCD may offer a workaround. A number of cellular mechanisms allow for stochastic phenotype switching in microbes, including phase variation^30^, contingency loci^44^, positive feed-back loops in gene expression^45^ and epigenetic phenotypic ‘memory’ due to the slow turnover rate of cellular constituents^46,47^. One of the elegant aspects of PCD is that it may increase the efficacy of any of these mechanisms without necessarily incurring complex pleiotropic side-effects. Second, PCD may allow for tuning of switch rates to match different environments that a microbe encounters. For example, if phenotypic diversification is beneficial only in challenging environments, but is otherwise costly, then PCD linked to an environmental cue that is correlated with the challenging environment may be a simple way to plastically increase diversification rates (though, as above, evolution of phenotypically plastic switch rates, if possible, would be a superior solution). While PCD may not be the optimal path to generating phenotypic diversity, Darwinian evolution makes do with the heritable variation that is available to select upon, not that which is optimal, often generating complicated or inelegant solutions. Our modeling demonstrates that PCD may evolve in order to improve the efficacy of microbial diversification bet-hedging strategies, but it is important to keep key limitations of our work in mind: PCD will only increase diversity when the underlying diversification mechanisms are linked to reproduction, and it will always be more costly than the direct evolution of switching rates, though the latter may not always be possible.

Diversification through PCD may be an alternative to another common microbial bet-hedging mechanism: variable dormancy, in which only some cells of a clonal genotype become dormant^48,49^. Individuals that become dormant, be it through persistence or the formation of resting stages like spores or cysts, gain resistance to a broad spectrum of environmental insults, but pay a considerable opportunity cost if the environment becomes favorable to growth^50^. Our model suggests that another, subtler price of dormancy is the missed opportunity to acquire resistance to future environmental stresses, through increased phenotypic diversification. Intriguingly, there is evidence that PCD delays the onset of dormancy (sporulation) during the transition to stationary phase in some bacteria by supplying resources to starving survivors^50^. Although PCD by a subpopulation may conceivably benefit surviving cells in the event of an abrupt shift back to favorable conditions^51^, our model suggests that increased diversification may be another benefit of such population cycling.

The central findings of our model are amenable to experimental investigation. Specifically, we make three predictions that can be experimentally examined: (1) All else being equal, higher rates of PCD should increase phenotypic heterogeneity in microbes that stochastically diversify as a consequence of reproduction. (2) When phenotypic diversification acts as a bet-hedging strategy and risk is temporally uncorrelated (so that selection favors greater diversification^29^), PCD can be adaptive. (3) PCD creates a social dilemma by liberating resources that can be consumed by a PCD− competitor, causing PCD to be more beneficial when it occurs in a spatially-structured population.

Our model shares some similarities with a well-analyzed example of microbial altruistic suicide, the production of colicins^52,53^, known as the ‘suicide bomber’ game in evolutionary game theory^54–56^. Here, a small fraction of a bacterial genotype that possess the genes for colicin production express them, releasing the antibiotic into the environment by suicidal lysis. This altruistic suicide can be adaptive for the colicin-producing genotype, but, as with our model, is only beneficial when the environment is spatially structured^52^. Interestingly, when the genes for colicin production and resistance can be decoupled, this system may display non-transitive ‘rock paper scissors’ dynamics^53,56^. Here, the toxin-producing/resistant strain is beaten by a nonproducing but resistant competitor (it outgrows it), and once toxin producers are rare, the resistant strain is beaten by a susceptible nonproducerstrain (again, this outgrows the resistant competitor). The susceptible strain, however, can be beat by the original toxin-producer/resistant strain. Indeed, spatially-explicit agent-based game theoretic models are an exceptionally flexible and powerful approach for studying microbial social interactions^55^, and would be an ideal approach for further studying the role of PCD in the evolution of microbial bet-hedging strategies.

While our model considers the evolution of unicellular organisms, it may have some relevance for understanding the dynamics of cancer. Successful cancer lineages often overcome mechanisms inducing cellular apoptosis, which are widely believed to have evolved as an anti-cancer mechanism^57^. Retaining some residual PCD may be beneficial for cancer lineages (and thus detrimental to the host) if this increases generational turnover, increasing opportunities for stochastic differentiation, intra-tumor phenotypic heterogeneity^58^ and survival during unpredictable stresses such as chemotherapy.

Understanding the evolution of cellular suicide will require a plurality of approaches, informed by real-world ecology, backed by rigorous mathematical modeling and direct experimental investigation. We expect that there are many non-exclusive explanations for why microbes evolve PCD, but unfortunately few of the numerous hypotheses put forward in the literature have been mathematically investigated. Perhaps the main reason to model various hypotheses for PCD is to determine whether the conditions required for its evolution are permissive, or highly constrained. In this paper, for example, we find that high levels of PCD are only supported when the population is structured, but we also find that simple processes of death and clonal reproduction readily generate the necessary structure. While much work remains before we have a complete understanding of altruistic suicide, it is well worth the effort. Not only is it a topic of fundamental biological importance, it also has the potential to help generate novel therapeutic interventions^24,59^.

## Electronic supplementary material

Supplementary Information
