# Publisher Correction: Programmed cell death can increase the efficacy of microbial bet hedging

**DOI:** 10.1038/s41598-018-23599-6

**Published:** 2018-04-19

**Authors:** Eric Libby, William W. Driscoll, William C. Ratcliff

**Affiliations:** 1 Santa Fe Institute, Santa Fe, NM 87501 USA; 2 Ecology, Evolution and Behavior, University of Minnesota, Minneapolis, MN 55108 USA; 3 School of Biological Sciences, Georgia Institute of Technology, Atlanta, GA 30332 USA

Correction to: *Scientific Reports* 10.1038/s41598-017-18687-y, published online 18 January 2018

The original version of this Article contained an error in the title, where the phrase “bet hedging” was incorrectly given as “bet -hedging”. This has now been corrected in the PDF and HTML versions of the Article.

