# Supplementary material for: Programmed cell death can increase the efficacy of microbial bet hedging
Source: Sci Rep. 2018 Jan 18;8:1120. doi: 10.1038/s41598-017-18687-y (PMC5773525; doi:10.1038/s41598-017-18687-y)
Supplement: Supplementary file 1 — Supplementary Information [file 41598_2017_18687_MOESM1_ESM.pdf]

Programmed cell death can increase the efficacy of microbial bet hedging [Supplementary material]

Eric Libby[1], William W. Driscoll[2], and William C. Ratcliff[3,*]

[1]Santa Fe Institute, Santa Fe, NM 87501, USA
[2]Ecology, Evolution and Behavior, University of Minnesota, Minneapolis, MN 55108, USA
[3]School of Biology, Georgia Institute of Technology, Atlanta, GA 30332, USA
[*]william.ratcliff@biology.gatech.edu

Supplementary material

Here, we consider a continuous model in which there is an explicit rate of growth (.01, modulated by proximity to a carrying capacity N) and a constant rate of death independent of PCD (.001). The basic model assumes an arbitrary number n of strains where the dynamics of any given strain i is described by the following set of equations:

$$
\begin{aligned}
K &= 0.01\left(N-\sum_{j=1}^{n}(A_j+B_j+D_j)\right) \qquad (1)\\
\frac{\partial A_i}{\partial t} &= K\left((1-p_i)A_i+p_iB_i\right)+rD_i/2+\sum_{\substack{j=1\\ j\neq i}}^{n}\left(\frac{(1-r)}{2(n-1)}D_j\right)-c_iA_i-.001A_i\\
\frac{\partial B_i}{\partial t} &= K\left((1-p_i)B_i+p_iA_i\right)+rD_i/2+\sum_{\substack{j=1\\ j\neq i}}^{n}\left(\frac{(1-r)}{2(n-1)}D_j\right)-c_iB_i-.001B_i\\
\frac{\partial D_i}{\partial t} &= c_iA_i+c_iB_i
\end{aligned}
$$

The A_i and B_i terms correspond to the amount of A and B for strain i while the D_i term is the PCD dead pool that are replaced according to the structure parameter r.

We use this continuous model to explore what happens if populations are not assured of reaching carrying capacity. We use an iterative simulation in which during each iteration there is a chance a disaster occurs (with probability 0.1) that randomly removes all A or B phenotypes. We then solve the continuous model for $t = .025$ time units which results in populations that sometimes reach carrying capacity and sometimes do not. We use this algorithm to simulate a PCD strain invading a non-PCD population at 1% relative frequency (see Figure S1). We find that despite the low initial abundance of the PCD strain and the population not being restored reliably to carrying capacity ($N = 10,000$), the PCD strain can still invade and reach a majority of the population.

Figure S2 shows the effect of the time duration of growth on the ability of the PCD strain to invade. When there is very little population growth in between disasters it can delay or even prevent the PCD strain from invading. However, when the growth duration is increased the PCD strain can invade faster, i.e. in fewer number of rounds of growth/disaster.

Now, we consider an evolutionary simulation in which populations contain multiple strains with evolvable rates of PCD. We use the same algorithm as generated Figure S1 (also $r = .95$) except we make a small addition that a new mutant can appear at the start of an iteration with probability .0001. With probability .25, the new mutant will have a PCD rate equal to 0. Otherwise, the PCD rate is sampled randomly from 10^{-3} to 1 according to $10^{\mathrm{unif}(-3,0)}$. The probability of switching is fixed at .0001. We also relax the assumption that disasters completely remove a phenotype and instead assume that they remove 95% of the targeted phenotype. The results are shown in Figure S3.

Finally, we consider a simulation in which the probability of switching can also evolve (see Figure S4). When a mutation occurs it can either alter the PCD rate or the probability of switching. If the PCD rate is altered then as before with probability .25, the new mutant will have a PCD rate equal to 0. Otherwise, the PCD rate is sampled randomly from 10^{-3} to 1 according to $10^{\mathrm{unif}(-3,0)}$. If the mutation affects the rate of switching then the new rate is sampled randomly from 10^{-4} to 1 according to $10^{\mathrm{unif}(-4,0)}$. In general we find that this evolutionary simulation has two potential courses. Since there is only a fitness benefit to switching faster, if the switching rate evolves to be higher first then we do not observe any increase in PCD rate. If instead PCD rate increases first then eventually there will be a mutation that increases the rate of switching which will in turn make PCD less beneficial (see Figure S4 for a sample trajectory).

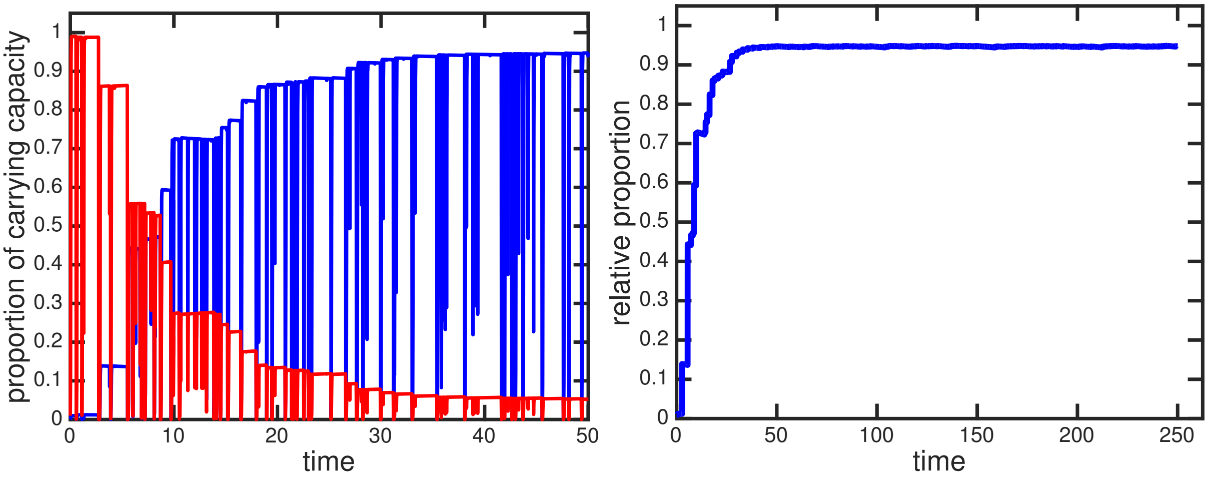

Figure S1. Competition between PCD and non-PCD strain in continuous time model. (Left) The resident non-PCD strain (red) is invaded by an initially rare 1% PCD strain (blue) with apoptosis rate $c = .01$. Both populations switch with $p = .01$ and $r = .95$. Every $t = .025$ a disaster may occur with probability .1. As a result populations frequently are not restored to carry capacity. (Right) The relative proportion of the population that is PCD rises to close to 95% where it remains for the duration of the simulation.

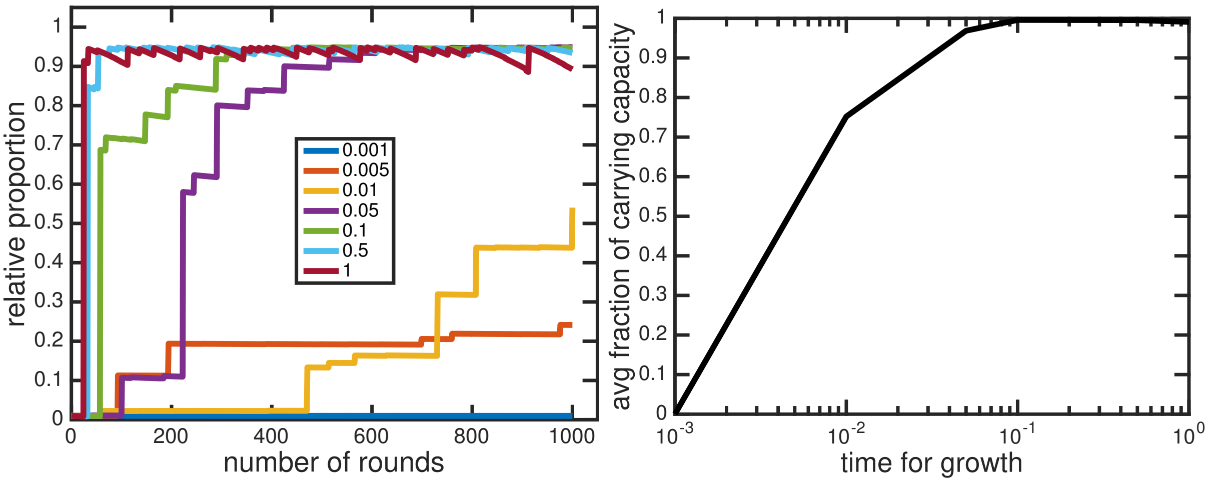

Figure S2. The effects of the duration of growth between disasters. (Left) The average proportion of the PCD strain is shown as a function of the number of growth rounds for different durations of growth. For very short growth periods, the PCD strain cannot invade. As the growth durations increase the PCD strain can invade faster and faster. (Right) The average population size (relative to the carrying capacity) is shown as a function of the growth durations. In between a growth duration of .01 and .1 the population is below carrying capacity but the PCD strain can still invade.

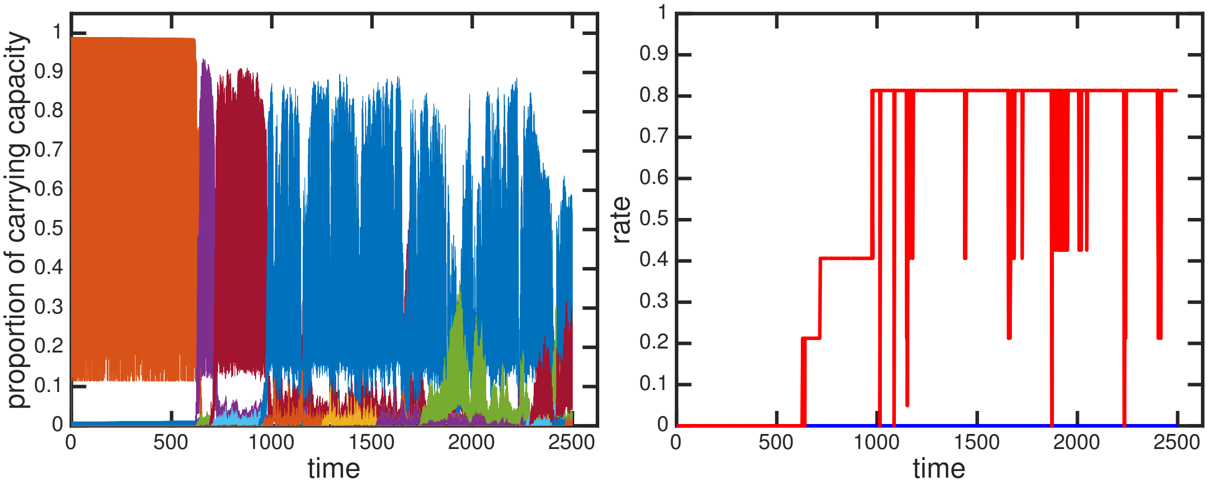

Figure S3. Evolutionary simulation in which the rate of PCD is an evolvable trait. (Left) The population proportion of different lineages is shown as a function of time. The different colors correspond to genotypes with different rates of PCD. Despite the non-PCD strain starting at 99% of the population it is replaced by different PCD strains. (Right) The PCD rate of the most abundant strain is shown as a function of time. The PCD rate evolves to be quite high but the plot on the left shows that there is also coexistence with other PCD strains.

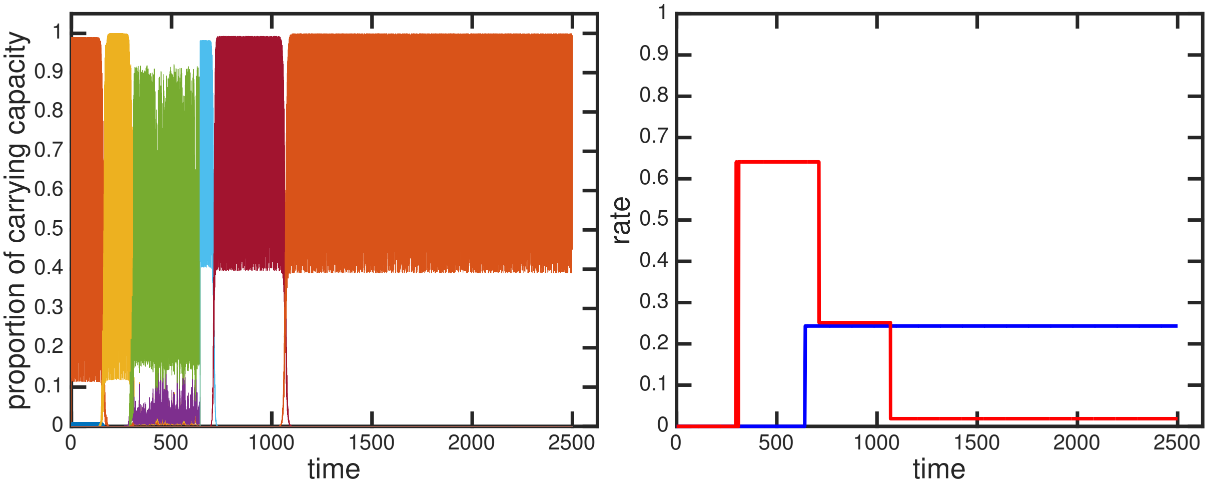

Figure S4. Evolutionary simulation in which the rates of PCD and switching are evolvable traits. (Left) The population proportion of different lineages is shown as a function of time. The different colors correspond to strains with different rates of PCD and/or switching. Despite the non-PCD strain starting at 99% of the population it is replaced by different strains that ultimately reduce the population fluctuations. (Right) The PCD rate (red) and rate of switching (blue) of the most abundant strain is shown as a function of time. The PCD rate evolves to to be higher at first but once the probability of switching increases there is no longer a benefit to PCD and its rate evolves to be lower.
